# Characteristics of the Contrast Enema Do Not Predict an Effective Bowel Management Regimen for Patients with Constipation or Fecal Incontinence

**DOI:** 10.7759/cureus.745

**Published:** 2016-08-23

**Authors:** Jordan Huber, Douglas C Barnhart, Shawn Liechty, Sarah Zobell, Michael D Rollins

**Affiliations:** 1 School of Medicine, University of Utah School of Medicine; 2 Division of Pediatric Surgery, Primary Children's Medical Center; 3 Department of Surgery, Primary Children's Medical Center

**Keywords:** bowel management, fecal incontinence, constipation, hirschsprung’s disease, anorectal malformations, idiopathic constipation, contrast enema

## Abstract

*Background*: A bowel management program using large volume enemas may be required for children with anorectal malformations (ARM), Hirschsprung’s disease (HD), severe medically refractive idiopathic constipation (IC), and other conditions. A pretreatment contrast enema is often obtained. We sought to determine if the contrast enema findings could predict a final enema regimen.

*Methods*: A retrospective review was performed at a tertiary care children’s hospital from 2011 to 2014 to identify patients treated with enemas in our bowel management program. Patient characteristics, contrast enema findings (including volume to completely fill the colon), and final enema regimen were collected.

*Results*: Eighty-three patients were identified (37 ARM, 7 HD, 34 IC, and 5 other). Age ranged from 10 months to 24 years, and weight ranged from 6.21 kg to 95.6 kg at the time bowel management was initiated. Linear regression showed contrast enema volume was of limited value in predicting effective therapeutic saline enema volume (R^2 ^= 0.21). The addition of diagnosis, colon dilation, and contrast retention on plain x-ray the day after the contrast enema moderately improved the predictive ability of the contrast enema (R^2 ^= 0.35). Median final effective enema volume was 22 mL/kg (range: 5 - 48 mL/kg).

*Conclusions*: We were unable to demonstrate a correlation with contrast enema findings and the effective enema volume. However, no patient required a daily enema volume greater than 48 mL/kg to stay clean.

## Introduction

Children who have undergone surgery for Hirschsprung’s disease (HD), anorectal malformations (ARM), and sacrococcygeal teratomas (SCT), or those who have severe medically refractive idiopathic constipation (IC) or spine or spinal cord abnormalities may suffer from fecal incontinence or constipation [1-4]. An individualized and comprehensive bowel management program is often required in order to keep the patient clean and in normal underwear [5]. Large-volume saline enemas are one of the treatment modalities used for bowel management with the goal of mechanically emptying the colon daily to avoid fecal accidents.

A pretreatment contrast enema is obtained at the start of bowel management to gain information regarding the diameter and length of the colon, stool burden, and provide catharsis. Providers may then use these findings to estimate the starting enema volume. During the initial week of bowel management, the patient’s clinical and radiographic responses to the enema are assessed daily. In most cases, the starting enema regimen is changed multiple times until an effective enema is discovered by trial and error. The treatment plan is considered successful when the abdominal radiograph is clear of stool in the rectum and left colon and the child has had no soiling.

Finding an effective enema regimen can be frustrating for the patient, the family, and the treating team. The bowel management program is very time-consuming, which may become extended if it is being conducted by a sole provider. The aim of this study was to determine if the contrast enema findings could predict a final enema regimen in order to simplify the trial and error process of bowel management.

## Materials and methods

**Figure 1:**
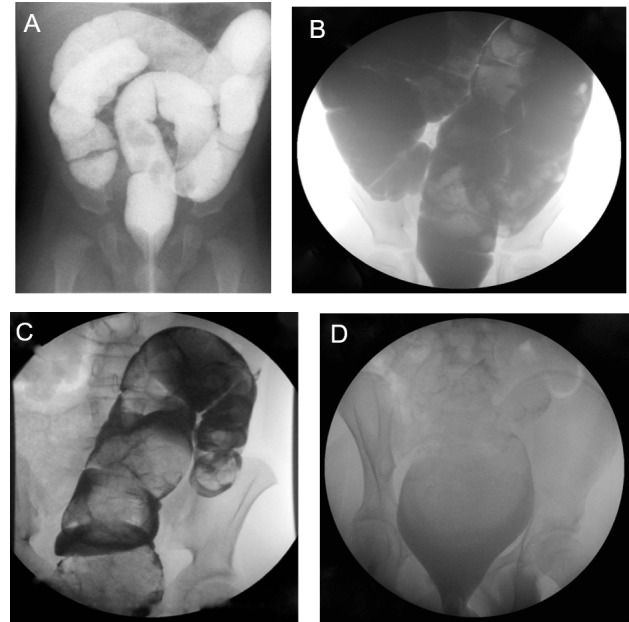
Contrast Enema Findings A = normal caliber, non-dilated colon; B = global dilation; C = rectosigmoid dilation; D = rectal dilation.

A retrospective review was conducted at our tertiary care children’s hospital from 2011 to 2014 with IRB approval from the University of Utah, approval #74392. Waiver of consent was obtained from IRB for this retrospective review. Children enrolled in the bowel management program at our colorectal center were identified. Patients managed with enemas were selected for further review. Large-volume saline enemas were selected on an individual basis to manage medically refractive constipation, fecal incontinence, and pseudo-incontinence. Patient characteristics included diagnosis, age, and weight at initiation of bowel management. Radiographic observations included colonic motility, which was inferred from retained contrast on follow-up x-ray the day after the contrast enema, redundancy and dilation of the colon, and contrast volume required to fill the entire colon during the study. The colon was characterized as non-dilated, dilated rectum, rectosigmoid dilation, or global dilation (Figure 1). For analysis, patients with rectal dilation were combined with the rectosigmoid dilation group. Treatment was successful if the abdominal radiograph was clear of stool in the rectum and left colon, and the child had no soiling within one week of starting the bowel management program. Complete records, including the final enema regimen with appropriate follow-up, were available for 94 patients.

Descriptive statistics were performed to summarize patient characteristics and enema findings. Contrast enema volume to completely fill the colon was assessed for its ability to predict the final effective therapeutic enema volume. This was done using linear regression with using SAS 9.2. Models were evaluated to examine both total contrast volume (mL) and contrast volume by weight (mL/kg). Additional models were created including diagnosis, colon dilation, and contrast retention on plain x-ray the day after the contrast enema. R^2^ was used to assess predictive values of the models.

## Results

Ninety-four patients with complete records and follow-up were identified. Eight patients were non-compliant with the program, and three patients were unsuccessful (88% success rate). Age at initiation of bowel management for the 83 patients ranged from 10 months to 24 years, and males comprised 57% of the study group. Diagnoses included anorectal malformation (37), Hirschsprung's disease (7), idiopathic constipation (34), sacrococcygeal teratoma (1), and spina bifida (4). The level of aganglionosis for patients with Hirschsprung disease and ARM types are listed in Table 1.

**Table 1:** Hirschsprung's Disease Level and Anorectal Malformation Type

Hirschsprung Level	# of Patients		ARM Type	# of Patients
Rectum	2		Cloaca	4
Rectosigmoid	3		Bladder Neck Fistula	3
Sigmoid	2		Perineal Fistula	4
Total	7		Rectal Atresia	3
			Rectal Stenosis	2
			Rectoprostatic Fistula	4
			Rectovaginal Fistula	7
			Rectovestibular Fistula	7
			Rectourethral Bulbar Fistula	2
			Unknown	1
			Total	37

Fifty-four percent of the patients were felt to have colonic hypomotility based on radiographic imaging. Contrast enema volumes (mL) ranged from 50 to 1500 for patients weighing < 20 kg; 150 to 2100 for patients 20 kg to 50 kg; 500 to 3300 for those weighing > 50 kg. Median volume of contrast to fill the colon was 38 mL/kg (range: 4 - 87 mL/kg). Patterns of colonic dilation are summarized in Table 2.

**Table 2:** Character of the Colon on Contrast Enemas ARM = Anorectal Malformation, HD = Hirschsprung’s Disease, IC = Idiopathic Constipation, SCT = Sacralcoccygeal Teratoma. Rectosigmoid dilation includes patients with rectal dilation and megarectum.

Diagnosis	Non-Dilated	Rectosigmoid Dilation	Global Dilation	Total
ARM	23	14	0	37
HD	5	1	1	7
IC	22	11	1	34
Spina bifida	3	1	0	4
SCT	0	1	0	1
Total	53	28	2	83

The final effective saline enema volume (mL) ranged from 200 to 600 for patients < 20 kg (median: 500 mL), 300 to 1000 for 20 kg to 50 kg (median: 600 mL), and 500 to 1000 for > 50 kg (median: 675 mL). Median final effective enema volume was 22 mL/kg (range: 5 - 48 mL/kg). The addition of glycerin or castile soap to the enema was variable. Linear regression demonstrated that the contrast enema volume was of limited value in predicting the final effective saline enema volume (R^2 ^= 0.21). The addition of patient diagnosis, the pattern of colonic dilation, and presumed hypomotility moderately improved the predictive ability of the contrast volume on the therapeutic enema volume R^2 ^= 0.35).

The overall success rate was 88%. The median length of time to determine the effective enema regimen was six days with an interquartile range of three days. The median length of follow-up was 20 months with an interquartile range of 28 months.

## Discussion

Successful bowel management is dependent on finding an effective enema volume to cause prompt and complete evacuation of the distal colon. This is typically done empirically with on-going adjustments to identify the correct volume. If the final volume could be predicted by contrast enema characteristics, it is possible that the final effective volume could be determined more quickly using fewer resources and causing less frustration for patients and families. Unfortunately, we were not able to demonstrate this. Rather, contrast enema results (volume to fill the colon and secondary characteristics) accounted for only 35% of the variability seen in the final effective enema volumes at one week. While the contrast enema was not predictive of the effective enema volume at the end of the bowel management week, we did not examine the ability of the contrast enema findings to predict the effective enema volume past one week.

This retrospective study is the first providing data on the relationship between pretreatment contrast enema findings and the therapeutic enema volume used for bowel management. A management algorithm for fecal incontinence in patients with a history of anorectal malformation, Hirschsprung's disease, or spina bifida using large volume enemas has been previously reported [5]. However, the authors recommend a saline volume that ranges widely. Furthermore, the findings of the contrast enema were not used to guide enema therapy in that study. Other studies in patients with varying diagnoses recommend enemas of 100 mL-200 mL. It has been recommended that individuals who have a non-dilated colon and diarrhea receive a lower volume than individuals with a dilated colon and constipation [2, 5-7]. Another recommendation has been made to start patients at a volume of 500 mL for incontinence and increase the volume up to 1000 mL, if necessary [4].

An important finding in our study is that no patient required a daily enema volume greater than 1000 mL to stay clean and most required much less. As might be expected, we found a trend toward larger saline enema volumes in larger patients. Unexpectedly, we found that the volume of contrast used for the contrast enema did not predict the final therapeutic saline enema volume. Furthermore, including the patient’s diagnosis, the pattern of colonic dilation and motility did not improve the predictive ability of the contrast enema volume in determining an effective bowel management regimen. We generally start patients ≤ 30 kg on a saline enema volume of 20 mL/kg and add irritants (soap) as needed. For patients > 30 kg, we will arbitrarily start at 600 mL of saline. The average therapeutic saline enema in our entire cohort was 23 mL/kg. The therapeutic saline volume for the majority of our patients > 20 kg was 600 mL.

Bowel management can be difficult to achieve in individuals with HD, ARM, SCT, and IC. Enemas are often used to help maintain continence. In our bowel management program, we had an 88% success rate out of 94 patients in determining the therapeutic saline enema volume. It can take substantial time and resources to determine the therapeutic enema volume. It may also be frustrating for patients and families. The aim of this study was to provide guidelines to reduce the time it takes to determine an effective enema.

Limitations of this study include the retrospective nature and the 8% non-compliance rate. One might argue that our patients with Hirschsprung's disease and ARMs with a good prognosis for bowel control (perineal fistula, vestibular fistula, rectal atresia, or urethral fistula) did not need enemas for bowel management. While it is our goal to manage constipation in these patients with stimulant laxatives, we will initiate enema therapy in patients who have never been toilet trained or if the rectum is severely dilated. A laxative trial is then attempted biannually or annually as desired by the patient and family. Finally, we did not examine the predictive value of the contrast enema in our patients who require laxatives for the management of severe constipation.

## Conclusions

Contrast enema findings do not correlate with the final effective enema in patients enrolled in our bowel management program. We recommend starting patients ≤ 30 kg on a saline enema volume of 20 mL/kg and add irritants (soap) as needed. For patients > 30 kg, we recommend arbitrarily starting at a 600 mL saline enema.
